# Predicting freshwater biological quality using macrophytes: A comparison of empirical modelling approaches

**DOI:** 10.1007/s11356-024-35497-8

**Published:** 2024-11-21

**Authors:** Daniel Gebler, Pedro Segurado, Maria Teresa Ferreira, Francisca C. Aguiar

**Affiliations:** 1 Department of Ecology and Environmental Protection, Poznan University of Life Sciences, Wojska Polskiego 28, 60-637 Poznan, Poland; 2 Forest Research Centre, Associate Laboratory TERRA, School of Agriculture, University of Lisbon, Lisbon, Portugal

**Keywords:** Macrophytes, Bioindication, River assessment, Artificial neural networks, Linear regression, Boosted regression trees

## Abstract

Difficulties have hampered bioassessment in southern European rivers due to limited reference data and the unclear impact of multiple interacting stressors on plant communities. Predictive modelling may help overcome this limitation by aggregating different pressures affecting aquatic organisms and showing the most influential factors. We assembled a dataset of 292 Mediterranean sampling locations on perennial rivers and streams (mainland Portugal) with macrophyte and environmental data. We compared models based on multiple linear regression (MLR), boosted regression trees (BRT) and artificial neural networks (ANNs). Secondarily, we investigated the relationship between two macrophyte indices grounded in distinct conceptual premises (the Riparian Vegetation Index — RVI, and the Macrophyte Biological Index for Rivers — IBMR) and a set of environmental variables, including climatic conditions, geographical characteristics, land use, water chemistry and habitat quality of rivers. The quality of models for the IBMR was superior to those for the RVI in all cases, which indicates a better ecological linkage of IBMR with the stressor and abiotic variables. The IBMR using ANN outperformed the BRT models, for which the r-Pearson correlation coefficients were 0.877 and 0.801, and the normalised root mean square errors were 10.0 and 11.3, respectively. Variable importance analysis revealed that longitude and geology, hydrological/climatic conditions, water body size and land use had the highest impact on the IBMR model predictions. Despite the differences in the quality of the models, all showed similar importance to individual input variables, although in a different order. Despite some difficulties in model training for ANNs, our findings suggest that BRT and ANNs can be used to assess ecological quality, and for decision-making on the environmental management of rivers.

## Introduction

Macrophytes are an important group of freshwater biota that commonly include vascular plants (ferns and angiosperms), bryophytes (mosses and liverworts) and macroscopic algae. These photosynthetic organisms are major primary producers and essential for several key processes, such as carbon and nutrient cycling and air–water-sediment exchanges (Haslam 1987). Macrophytes contribute to habitat creation, supporting other aquatic biota as refugia, nurseries and food sources and providing numerous ecosystem services (e.g. Gurnell et al. 2016; O’Hare et al. 2018).

Macrophyte species display consistent and stable responses to environmental change, especially concerning nutrient enrichment, sediment loading and hydrologic alterations (Hering et al. 2010; Aguiar et al. 2014 and references therein). In addition, the existence of distinct biogeographical regions across the world and Europe showing high homogeneity of aquatic plant species are recognised, such as in the Central-Baltic region or the Mediterranean basins (Bonada and Resh 2013; Murphy et al. 2019). These characteristics contributed to their inclusion in freshwater bioassessment systems worldwide and as biological quality elements, which are key indicators of the health of aquatic ecosystems, for implementing the European Water Framework Directive (WFD; Birk et al. 2012; Feio et al. 2021).

Highly seasonal and temporary rivers have few spontaneous submerged and truly aquatic macrophytes (hydrophytes) (Ferreira et al. 2002). For this reason, a line of research using both aquatic and riparian plants for biomonitoring has emerged, yielding indices and models applicable to various Mediterranean countries (e.g. Aguiar et al. 2009; Papastergiadou et al. 2016). Nevertheless, using aquatic flora (mostly hydrophytes and emergent species) is mandatory under the WFD requirements, and official indices for Mediterranean rivers are grounded in bioindication (Haury et al. 2006). Bioassessment in Southern Europe using macrophytes has been hampered by difficulties in reference data availability and the unknown effect of the interacting stressors on plant communities (Aguiar et al. 2014; Dodkins et al. 2012). Moreover, multiple pressures impact European rivers due to organic pollution inflow via adjacent agricultural lands, hydrologic and geomorphologic alterations, damming and channelisation. The Mediterranean region is widely considered to be highly impaired by water scarcity, which becomes more challenging in the context of climate change, with a projected decrease of 30% in annual mean precipitation (Bonada and Resh 2013; Feyen et al. 2020). Understanding how these different stressors interfere with aquatic organisms, including macrophytes, is essential for developing effective methods to assess and monitor the ecological status of aquatic ecosystems (Hering et al. 2010; Polst et al. 2022). This involves researching innovative assessment systems and advanced analytical tools for enhanced data analysis (e.g. Hering et al. 2018; Rolim et al. 2023).

Regression models are recognised as being valuable tools in management, environmental decision-making, evaluation and ecosystem protection for environments exposed to multiple pressures (Lewis et al. 2021; Park and Lek 2016). They can be used to solve intricate relationships among ecosystem components and to predict their condition in response to changing environmental factors (Tiyasha et al. 2020). In recent decades, various predictive methods have rapidly developed and can be successfully used for ecological modelling (Elith et al. 2008; Poisot et al. 2016; Provata et al. 2008). These methods are derived from simple linear and non-linear models. Moreover, more complex methods are used, such as neural networks, genetic algorithms, Bayesian networks, regression trees or random forests (Zhang et al. 2015). The different new regression methods showed satisfactory quality concerning all major groups of aquatic biota, such as benthic macroinvertebrates, fishes, phytoplankton and macrophytes. Many authors have also presented comparisons between different regression methods. However, studies rarely refer to macrophyte indices, especially compared to other biological indices used in freshwater ecology and bioassessment (Tiyasha et al. 2020 and references therein).

Studies on the ecological assessment of freshwaters indicate that it is difficult to identify specific factors responsible for their degradation, which is especially problematic in multi-pressure situations. Additionally, methods designed to indicate specific degradation factors, a range of pressures or overall degradation have limited capacity to capture the effects of other significant stressors (Poikane et al. 2020). This complicates efforts to improve freshwater ecosystems’ ecological status (Carvalho et al. 2019). Therefore, the main goal of our study was to identify and hierarchise the importance of the main environmental factors affecting the ecological status as measured by two macrophyte-based indices widely used in Portugal: the Riparian Vegetation Index (RVI; Aguiar et al. 2009) and the Macrophyte Biological Index for Rivers (IBMR; Haury et al. 2006). Our research underscores the crucial role of these environmental variables in predicting macrophyte indices, highlighting their significance and relevance. The rationale for our study is to demonstrate the feasibility of using models in ecological status assessment, even when relying on older datasets or in situations where biological studies are lacking (such as those before WFD implementation). This also includes smaller water bodies where standard monitoring was not followed, but environmental variables (such as water quality parameters) are available or easily obtainable.

## Material and methods

### Site selection

**Fig. 1:**
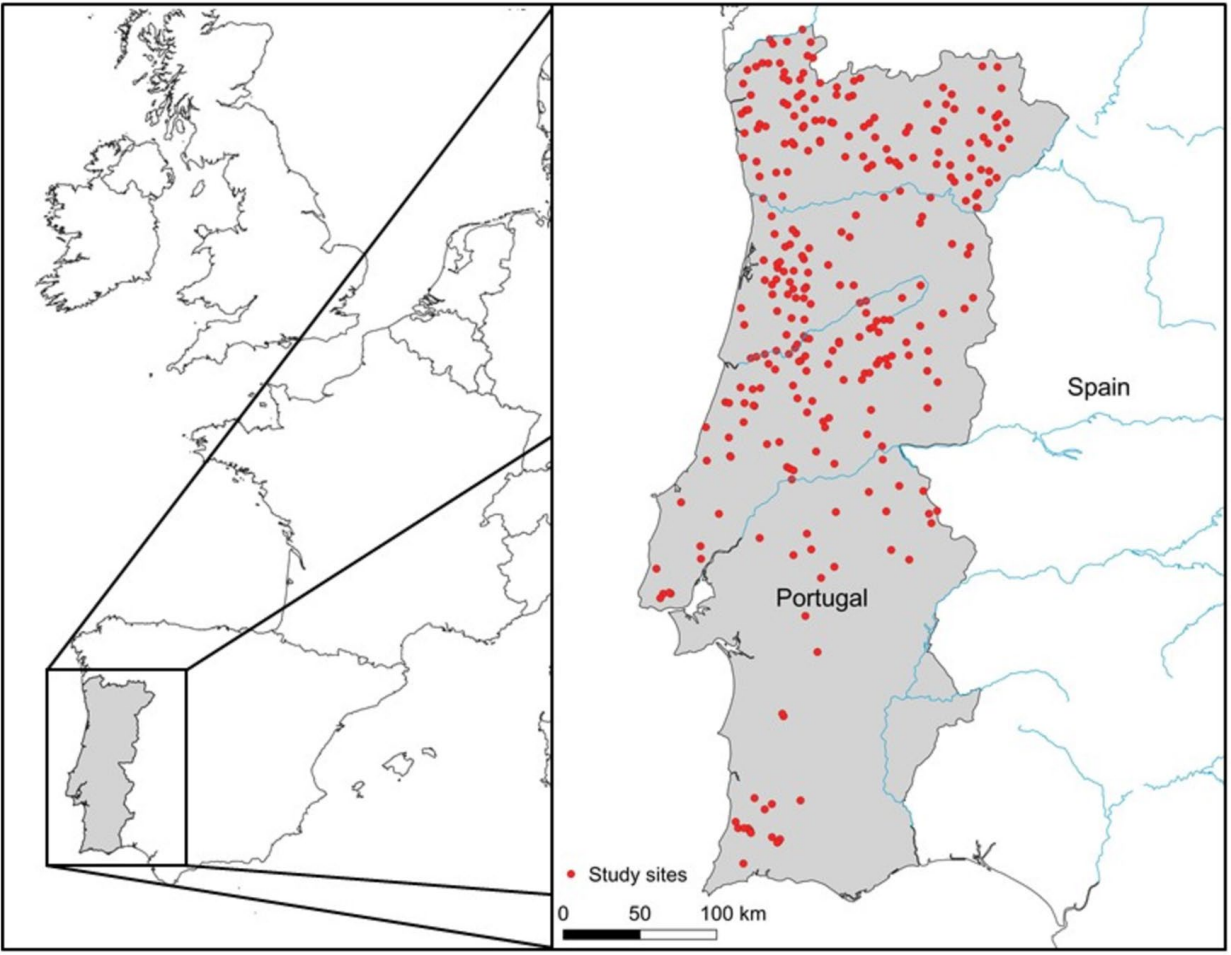
Map of the distribution of sampling sites (*n* = 292) in Portugal, SW Europe

The study concerns rivers and streams of mainland Portugal, on the western edge of the Iberian Peninsula, South Western Europe (Fig. 1). The country covers approximately 89,000 km^2^, with a large extension of the Atlantic Ocean coastline, and the influence of the Mediterranean Sea on the south region. Most of the study area has a temperate Mediterranean climate, with hot, dry summers and mild, wet winters. Inter-annual variability and multi-annual droughts are frequent and largely influence the distribution of macrophytes (Ferreira et al. 2002). The coastal area is densely populated and impaired by forestry, agriculture and industry, and the northern regions have a complex landscape of vineyards, orchards and small agricultural lands. South and inland areas have scattered settlements, extensive agricultural lands, Mediterranean scrubland and oak forests. Many rivers and streams in Southern regions have temporary streamflow regimes.

We used the macrophyte dataset (*n* = 402; 2004–2006; Portuguese Environmental Agency; https://www.apambiente.pt/dqa/macrofitos.html), developed for the WFD implementation in Portugal and the intercalibration of Mediterranean rivers and streams assessment systems. For modelling purposes, we defined a subset of sampling sites that agreed with the following criteria: (i) rivers and streams having perennial streamflow; (ii) abundance data on both aquatic and riparian plants; and (iii) availability of data on water chemistry, habitat quality and hydrology. We attained a final dataset of 292 sites, and a group of rivers in southern Portugal were removed from the database (Fig. 1). The main reason for site removal was the lack of aquatic macrophytes in streams with a temporary streamflow regime.

Sampling methods were based on the European standards EN14184:2003 and EN14996:2006 and followed a national protocol (Aguiar et al. 2014). Surveys were performed in late spring–early summer (May–June/July) in the in-stream part of the river channel that is submerged most of the year. The channel may be exposed temporarily under conditions of dry-water flow, usually in summer) or for extended periods under certain natural (climatic and geological) conditions. The sampling period allows for a survey of most macrophytes, including early flowering species (Ferreira et al. 2002). The field campaign started in the South as streams tend to dry out during summer. Sampling involves wading into the water and following a zigzag pattern upstream along the reach length, usually 100-m long sections of the river channel. In exceptional cases of high depth, surveys were conducted from one or both riverbanks. For the RVI, the surveys were performed both in-stream and on the riverbanks, including aquatic vegetation and riparian herbaceous and woody species (trees, shrubs, and lianas). Each taxon’s abundance was estimated as a percentage coverage of the surveyed transect. The data mainly included bryophytes and vascular plants. The Appendix presents a list of vascular hydrophytes (= aquatic species), helophytes (emergent species) and bryophytes (mosses and liverworts) that occurred in the active channel (Appendix Tables 3 and 4).

### Environmental variables

The nation-wide network abiotic database of rivers and streams allowed the selection of 24 environmental variables with standardised data characterising the conditions of monitoring sites (Table 1). The variables represent a group of environmental factors on a larger area scale, illustrating the geographical and climatic specificity of rivers and land use characteristics, as well as variables studied on-site, including three indices that characterise habitat quality, including two hydromorphological calculated by the application of the River Habitat Survey method (Raven et al. 1998), and nine variables characterise the water quality, e.g. water temperature, oxygen concentration, pH, orthophosphates and different forms of nitrogen (Table 1).

**Table 1:** Descriptive statistics of the explanatory variables

Variable	Range	*Mean* ± *SD*
**Geography and climate**		
Latitude (km in EPSG 20790 system)	93.6–323.7	209.1 ± 52.0
Longitude (km in EPSG 20790 system)	24.6–572.1	391.5 ± 129.5
Altitude (m)	3.0–1414.4	249.8 ± 237.5
Water thermal range (°C)	6.1–14.7	10.3 ± 1.3
Mean annual precipitation (mm)	489.0–2926.0	1156.6 ± 501.4
Mean annual runoff (mm)	75.0–2200.0	524.9 ± 389.4
Distance to source (km)	0.03–237.2	36.7 ± 43.1
Catchment area (km^2^)	1.1–5401.6	483.4 ± 963.7
**Land use**		
Natural areas in the catchment (%)	2.0–100.0	61.4 ± 26.0
Artificial areas in the catchment (%)	0.0–40.0	3.4 ± 5.6
Extensive agriculture in the catchment (%)	0.0–76.0	6.1 ± 10.2
Intensive agriculture in the catchment (%)	0.0–100.0	30.2 ± 24.3
**Habitat quality**		
Habitat Quality Assessment ( −) (Raven et al. 1998)	14.0–64.0	41.8 ± 8.5
Habitat Modification Score ( −) (Raven et al. 1998)	0.0–67.0	11.4 ± 11.7
Riparian Forest Quality Index ( −) (Munné et al. 2003)	0.0–100.0	57.4 ± 25.5
**Water quality**		
Water temperature (°C)	5.7–29.2	16.0 ± 3.8
Dissolved oxygen concentration (mg O_2_/L)	2.1–17.5	9.5 ± 2.1
pH ( −)	5.0–9.0	7.0 ± 0.7
Conductivity (µS/cm)	9.2–1388.0	183.9 ± 243.1
Alkalinity (mg HCO_3_^2−^/L)	1.5–442.0	57.2 ± 77.2
Total suspended solids (mg/L)	0.0–88.0	9.0 ± 12.7
Nitrates (mg NO_3_^–^/L)	0.01–34.2	3.4 ± 4.7
Ammonia (mg NH_4_^+^/L)	0.01–13.0	0.3 ± 1.0
Orthophosphates (mg PO_4_^3–^/L)	0.01–6.0	0.2 ± 0.5

### Macrophyte-based indices

The IBMR was first described by Haury et al. (2006) as an index for assessing water trophy and organic pollution. The IBMR was accepted as an official national method for classifying the ecological status of highly seasonal rivers of all EU Member States of the Mediterranean Geographical Intercalibration Group, except Slovenia (Aguiar et al. 2014). Following the adaptation of the WFD, the IBMR was tested by other Mediterranean countries, such as Turkey (Özbay et al. 2019) and Greece (Stefanidis et al. 2022). It is calculated using the following formula:

$$IBMR=\frac{\sum_{i=1}^{N}({CS}_{i}\times {E}_{i}\times {K}_{i})}{\sum_{i=1}^{N}({E}_{i}\times {K}_{i})}$$

where *i* — bioindicator taxon, *CS*_*i*_ — indicator value for the *i*-th taxon (0–20) expressing the preferred trophy level, *E*_*i*_ — stenoecy coefficient (weighting factor) of the *i*-th taxon, expressing ecological tolerance (1–3), and *K*_*i*_ — abundance of the *i*-th taxon (translated in 5 classes). It has been established that a minimum number of four bioindicator taxa is needed for a reliable IBMR calculation in Mediterranean rivers (Aguiar et al. 2014).

The RVI is a multimetric macrophyte-based index that uses the responses of structural and functional parameters of aquatic and riparian vegetation to global disturbance (Aguiar et al. 2009). The RVI has been widely used for environmental impact assessment purposes and general hydromorphological diagnostic of rivers and streams in mainland Portugal. It includes compositional metrics (e.g. cover and number of alien and endemic species) and functional metrics associated with life cycle and reproduction (e.g. proportion of perennial species) and with the trophic status (e.g. proportion of nitrophilous species). RVI uses a table of conversion of metric values into dimensionless values through a scale of three scores: 1 — poor quality, 3 — fair quality and 5 — good quality. Metric values were obtained using reference and non-reference site values for each parameter. The RVI for a site was obtained by the sum of the quality scores of all metrics, subtracted by the total number of metrics (for more details, see Aguiar et al. 2009).

### Modelling approaches

We tested three types of models: multiple linear regression (MLR), boosted regression trees (BRT) and artificial neural networks (ANNs). The dataset was randomly divided into two or three datasets used in different phases of model creation. Seventy percent of the cases were used in the training of models. The remaining 30% of cases were then used to validate the MLR and the BRT. ANN modelling consists of three phases: training, additional testing and validation. Therefore, the testing and validation datasets used 15% of cases each. Concerning the environmental variables dataset, we reduced their number and avoided collinearity between them using factor analysis (FA) (StatSoft Inc. 2017). The reduction of input variables in the model leads to fewer cases needed in the modelling process, simplifying the overall structure of models and eliminating redundant information without affecting the model’s error (Dormann et al. 2013).

Multiple linear regression is a classical statistical approach used in an enormous number of historical and current studies. It is a method based on the assumption of a linear relationship between one dependent variable and a set of independent explanatory variables (Olive 2017). In our study, we fitted candidate MLR models using all possible combinations of predictors. The final model selection was based on a multimodel inference procedure (Grueber et al. 2011) using the Akaike Information Criteria (AIC; Burnham and Anderson 2002). Models were ranked in increasing order of *AIC* values, and those with delta *AIC* < 2 (i.e. the absolute difference between the *AIC* of each model and the best approximating model below 2) were retained. The regression coefficients of the final model were then derived by averaging the estimated coefficients of the retained models (Burnham and Anderson 2002). MLR models were fitted using R version 4.2.0 (R Core Team 2022), and the multimodel inference was performed with the MuMIn package for R (Bartoń 2023).

Boosted regression trees are additive regression models that make predictions by combining decisions from a sequence of models, in this case, based on regression trees that seek to split data into increasingly homogeneous groups (Breiman et al. 1984). At each step of the learning process, each tree aims to explain the variation in data not explained by the previous tree until a certain level of predictive performance is achieved. Each regression tree is fitted using a random sample of observations, and each node within the tree is based on a random subset of variables. In this study, we fitted BRT models using the optimisation procedure implemented in the dismo package for R version 1.3–9 following the stepwise procedure recommended by Elith et al. (2008). To optimise the number of trees in each BRT model, we performed a stepwise process based on tenfold cross-validations using mean deviance to measure predictive performance. The tree complexity, a parameter that controls the number of interactions among variables (i.e. the number of splits of individual trees), was set to two (pairwise interactions). A second parameter, the learning rate, which determines the contribution of each tree to the growing model, was set iteratively to ensure that at least 1000 trees were achieved after the stepwise process (Elith et al. 2008).

Artificial neural networks are a type of deep learning algorithm that consists of layers of interconnected nodes (artificial neurons) that can learn and extract features from data. ANNs use back propagation to update the weights and biases of the neurons in each layer, optimising the network to make better predictions. This study used the multilayer perceptron (MLP) type of ANN with a Broyden–Fletcher–Goldfarb–Shanno (BFGS) algorithm, available in STATISTICA 13 (StatSoft Inc. 2017). This type of network is trained with the supervised teacher technique called the delta rule. The ANN was previously recognised as a valuable method for modelling non-linear relationships (Park and Lek 2016) and was used in similar studies (Gebler et al. 2018; Krtolica et al. 2021; Rocha et al. 2017). A three-layer neural network was used in this study. The first layer included factors obtained in the FA. The hidden layer consisted of neurons ranging from (*2n*^*1/2*^ + *m*) to (*2n* + *m*), where *n* and *m* are the number of input and output neurons, respectively (Fletcher and Goss 1993). The out neuron was always one (IBMR or RVI).

Following recommendations (e.g. Park and Lek 2016), the application of statistical models was preceded by output data normalisation (min–max normalisation) to a range of 0.1–0.9. In factor analysis, the input variables were standardised by the autoscaling method (linear transformation carried out by scaling the values with *mean* = 0 and *variance* = 1).

The quality of each model (i.e. MLR, BRT and ANN) was evaluated using three parameters that are commonly used performance measures in similar studies (Hernandez‐Suarez and Nejadhashem 2018): coefficient of determination (*R*^2^, Eq. (1)) representing the amount of explained variance, the *r*-Pearson correlation coefficient (*r*, Eq. (2)) showing the fitness of data, and the normalised root mean square error (*NRMSE*, Eq. (3)) based on values of biological indices and modelled values.

1 $${R}^{2}=1-\frac{\sum_{i=1}^{n}{({y}_{i}-{y}_{i}{\prime})}^{2}}{\sum_{i=1}^{n}{({y}_{i}-{\overline{y} }^{i})}^{2}}$$

2 $$r=\frac{\sum_{i=1}^{n}({y}_{i}-\overline{y })({y}_{i}{\prime}-{\overline{y} }{\prime})}{\sqrt{\sum_{i=1}^{n}{({y}_{i}-\overline{y })}^{2}\sum_{i=1}^{n}{{(y}_{i}{\prime}-{\overline{y} }{\prime})}^{2}}}$$

3 $$NRMSE=\frac{\sqrt{\frac{\sum_{i=1}^{n}{\left({y}_{i}{\prime}-{y}_{i}\right)}^{2}}{n}}}{{y}_{\text{max}}-{y}_{\text{min}}}$$

where ${y}_{i}$ — *i*-th value of the output variable, $\overline{y }$ — mean value of the output variable, ${y}_{i}{\prime}$ — *i*-th value of the output variable derived from the model, ${\overline{y} }{\prime}$ — mean value of the output variable derived from the model, ${y}_{\text{max}}$ — maximum value of the variable, ${y}_{\text{min}}$ — minimum value of the variable and *n —* number of cases.

The variable importance measures (Wei et al. 2015) were computed to identify which environmental factors significantly influence the modelled values of the two macrophyte indices. The relative importance of predictor variables in MLR was assessed based on the variable effect sizes. In the case of BRT models, the relative importance of each predictor was estimated by averaging the number of times each variable was selected for splitting a tree and the squared improvement resulting from these splits. A sensitivity analysis was carried out for the ANNs. This analysis shows how much the model error would increase after removing a given variable. Higher values indicate greater importance of the variable in the model. To compare the variable importance derived by different models (MLR, BRT and ANN), in each case, the relative influence of each variable in each model was scaled to 100, with higher numbers indicating a higher contribution to the outputs (Elith et al. 2008).

## Results

Factor analysis (FA) enabled us to reduce the original set of 24 environmental variables to eight latent factors (Appendix Table 5). The first factor combines longitude, alkalinity and conductivity information, indicating geographical gradient and geology information. The second extracted factor refers to water quality in terms of nutrient content. The third factor was an emanation of the water body’s size, described by the catchment’s size and the associated distance from the source. The fourth factor represented hydrological and climatic conditions. The fifth factor indicated the habitat quality, encompassing the naturalness and modifications of the river channel and riparian zone. The next factor (6th) was related to different catchment land-use categories. The last two factors (7th and 8th) were related to the physicochemical quality of water (total suspended solids and pH) and oxygen conditions, respectively. The eight identified factors explained 71.4% of the variance in the environmental variables presented by the dataset. The acquired factors were subsequently used as explanatory (input) variables in all models for both IBMR and RVI indices.

### Modelling performance

The various models were developed to address specific needs and challenges within the study context. The multiple linear regression models resulted from averaging the two and four best approximating models for IBMR and RVI, respectively. The IBMR model included all the FA factors, and except factor 8; all regression coefficients were significantly different from 0 (*z*-test; *p* < 0.05). The model for RVI also included all the FA factors, and all were significantly different from 0, except for factors 7 and 8. The boosted regression tree models used Gaussian loss functions and consisted of a sequence of 1600 trees for IBMR and 1900 trees for RVI, with all eight predictors showing significant non-zero influence. The constructed neural network models had three layers and differed in the number of neurons in the hidden layer. The number of hidden neurons for the best network modelling IBMR was 7, and for RVI, it was 17. Logistic (IBMR) and logistic and exponential (RVI) functions were used as activation functions.

**Fig. 2:**
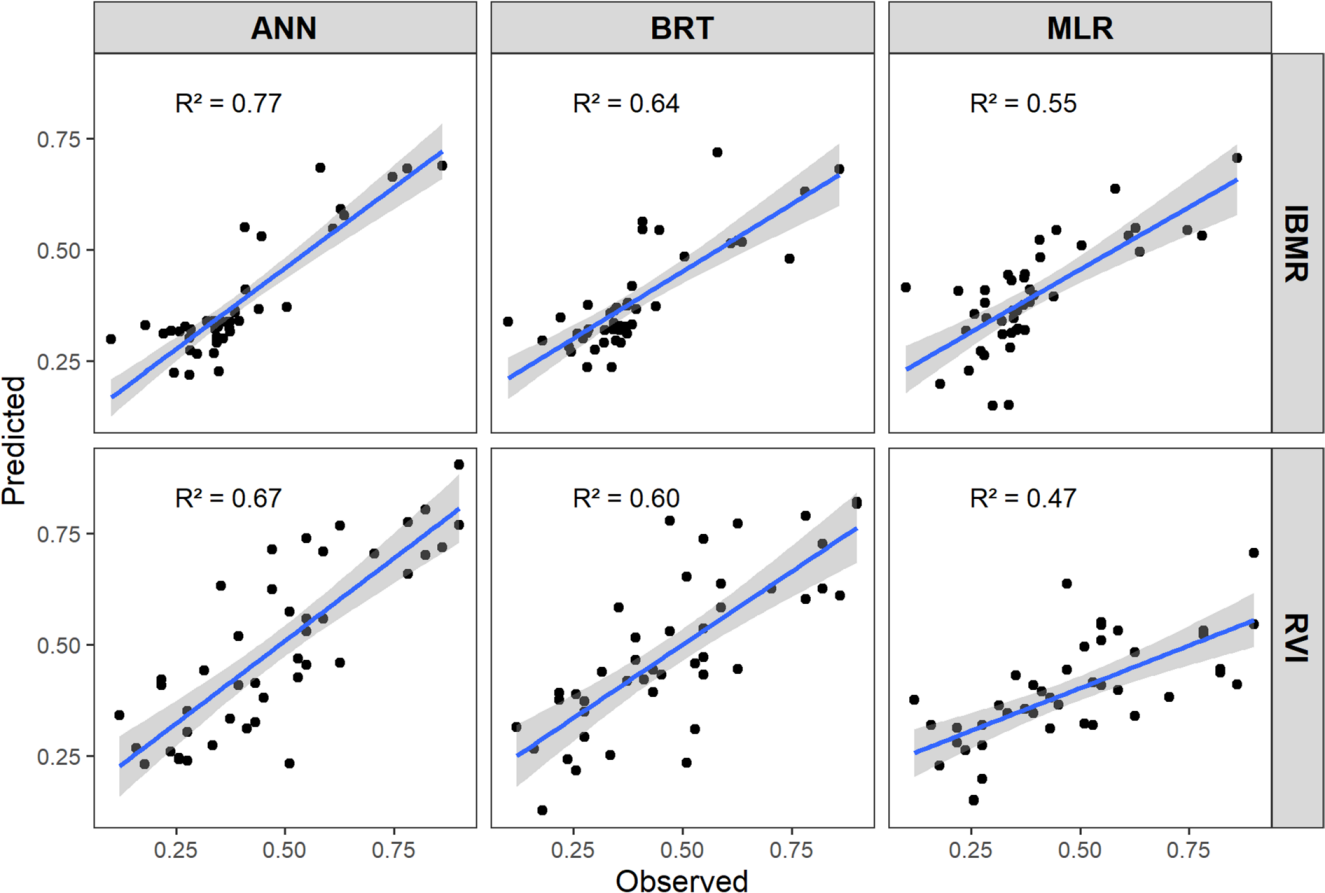
Values of IBMR and RVI (validation dataset) predicted and observed by different models

All methods used (MLR, BRT and ANN) for the IBMR prediction had an overall higher quality than the exact methods modelling the RVI. The analysis of model quality focused on evaluating the performance parameter values for the calibration-independent validation dataset. A comparison of the model quality of all models is presented in Table 2. The plot of the modelled against observed values of the macrophyte indices is presented in Fig. 2. It is noteworthy that, regardless of the modelled index, the hierarchy of the efficacy of the different regression methods remained consistent and was ranked as follows: ANN > BRT > MLR. The ANN revealed the strongest correlation between the predicted and observed values, showing consistent similarity. This model also explained the highest percentage of the variance of the modelled variables and had the lowest modelling errors. This was demonstrated for the IBMR model by the value of the coefficient of determination for the final validation dataset (0.77), the *r*-Pearson correlation coefficient (0.88), and the normalised mean squared error value, which did not exceed 10.0%. The same type of model for the RVI was of correspondingly lower quality (*R*^2^ = 0.67, *r* = 0.82, *NRSME* = 15.7%). It is also worth emphasising that the quality of ANN models was higher than BTR, but not much, while both models were clearly better than MLR.

**Table 2:** Quality statistics of the constructed ANNs

Index	Type of model	*r*			*NRMSE* (%)		
		Training	Testing	Validation	Training	Testing	Validation
IBMR	MLR	0.73	-	0.74	12.7	-	13.8
	BRT	0.94	-	0.80	6.5	-	12.3
	ANN	0.88	0.87	0.88	8.9	12.4	10.0
RVI	MLR	0.68	-	0.68	23.9	-	22.7
	BRT	0.88	-	0.78	12.8	-	17.1
	ANN	0.85	0.80	0.82	13.9	18.2	15.7

The prediction accuracy of BRT for both indices showed a diminished quality in the final validation procedure when compared to the ANN. Despite higher performance parameters during the training step, which marks the initial phase in model creation, BRT’s overall validation performance did not surpass ANN’s. Moreover, a consistent dependency emerged between the BRT models for the two indices, indicating that the prediction of the IBMR prediction was more accurate than that for the RVI. The values of the determination coefficient for the validation procedure were 0.64 and 0.60, and the fitness of the modelled and observed data determined by the *r*-Pearson correlation coefficient were 0.80 and 0.78, respectively. Moreover, the *NRMSE* values were 12.3% vs. 17.1%.

Both MLR models exhibited the lowest prediction quality and demonstrated subpar performance parameters. The coefficients of determination for the IBMR and RVI models were below 0.5, signifying that these models accounted for less than half of the variance in the modelled variables. Furthermore, a less accurate fit of the modelled data (*r* = 0.74 and 0.68, respectively) and higher errors (*NRMSE* 13.8 vs 22.7%) were obtained, again indicating a better model quality for the IBMR than the RVI.

### Variable importance measures

**Fig. 3:**
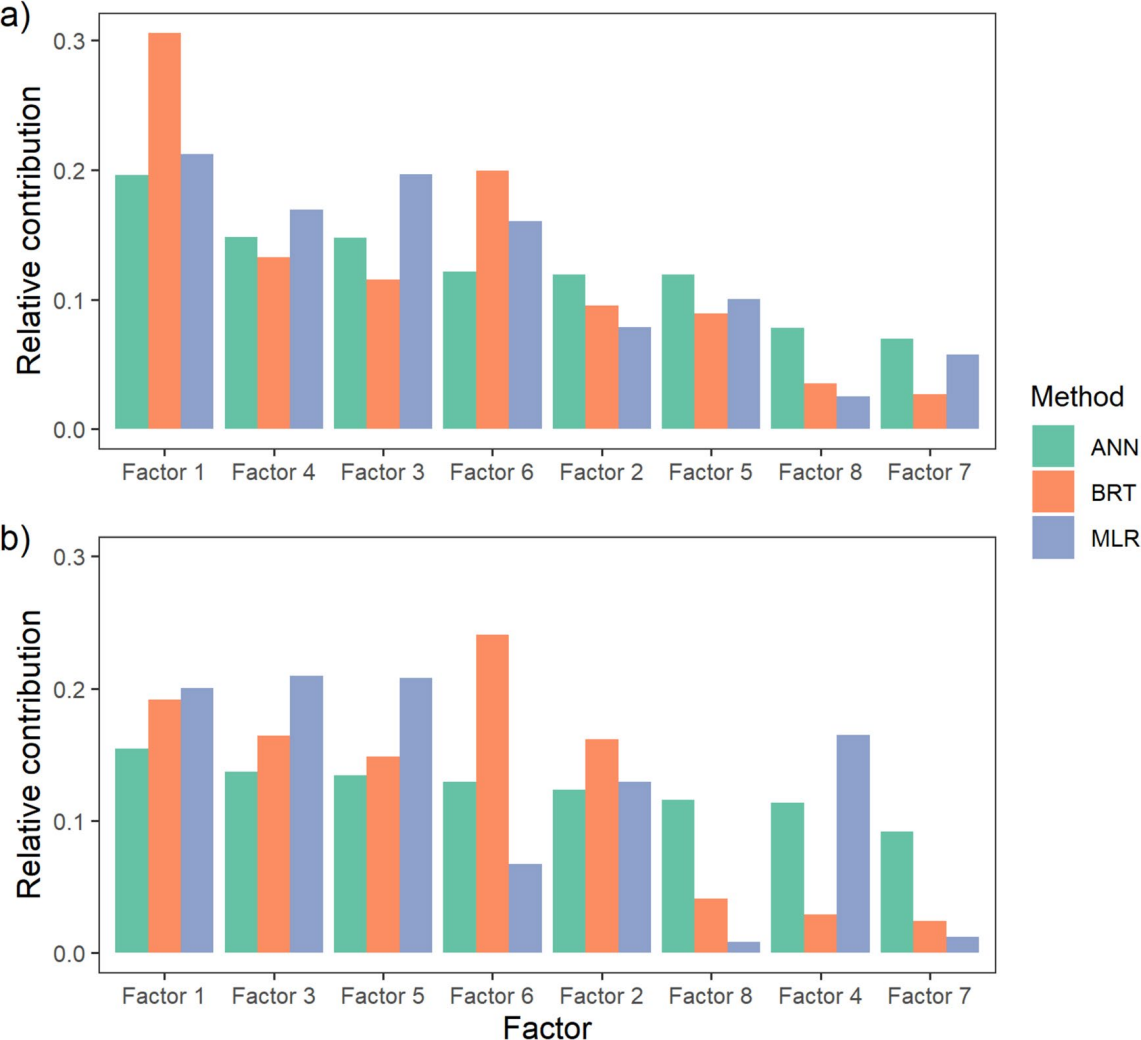
Relative contribution of input variables (factors derived in FA) in different IBMR (**a**) and RVI (**b**) models

The diverse modelling methods displayed similar importance to the individual input variables (Fig. 3) despite noticeable differences in the performance of particular models. In most cases, Factor 1, encompassing longitude and catchment geology, exerted the most significant influence on the modelled values of the IBMR index. This factor proved to be the most important in the ANN and BRT models. Hydrological and climatic conditions (Factor 4), water body size (Factor 3) and land use (Factor 6) were also crucial input factors in models, albeit with different ranking positions. The hydrological and climatic factors were second in the ANN and third in BRT, and the water body size factor was third (value very close to Factor 4) in the ANN and fourth in BRT. Land use emerged as the fourth most influential in the networks but appeared second in BRT. In addition, two other factors, nutrients (Factor 2) and habitat quality (Factor 5), also affected the modelling values of the IBMR, but their importance was relatively lower. The final order of the first six variables in the ANNs was as follows: Factor 1 > Factor 4 > Factor 3 > Factor 6 > Factor 2 > Factor 5, while in the BRT, it was as follows: Factor 1 > Factor 6 > Factor 4 > Factor 3 > Factor 2 > Factor 5. The six input parameters selected for MLR overlapped with the most relevant parameters for the ANN and BRT, and their order closely resembled that of the previous methods: Factor 1 > Factor 3 > Factor 4 > Factor 6 > Factor 2 > Factor 5. The other parameters, namely oxygen conditions, TSS and pH (Factors 8 and 7), were consistently ranked lower across all three methods, indicating their diminished importance in influencing the IBMR models.

In the case of the RVI, the various models displayed greater differences in the importance of specific factors compared to the IBMR (Fig. 3). Despite these differences between the indices, it is noteworthy that for the RVI, longitude and geology (Factor 1) were also essential parameters. Factors 3 and 6 were also important, encompassing water body size and land use, respectively, along with habitat quality (Factor 5) and nutrients (Factor 2). Similar to previous models for the IBMR, Factor 7 (TSS and pH) has little importance, and in contrast to the IBMR, Factor 4, i.e. hydrological and climatic conditions, although still significant for MLR, is not as crucial for ANN and BRT models.

## Discussion

The study showed that different modelling approaches can express and quantify the relationship between the macrophyte-based indices for ecological status assessment and a range of environmental variables. This underscores the potential of these models as practical tools for predicting the biological quality of water bodies that are not subject to direct monitoring. The accuracy of such predictions relies on carefully selecting the most suitable model and its associated environmental variables. The quality of modelling by artificial neural networks (ANNs), boosted regression trees (BRT) and multiple linear regression (MLR) can vary according to the specific goals and the dataset (units, size and quality). However, machine-learning techniques often improve prediction quality (Hernandez‐Suarez and Nejadhashem 2018; Mata et al. 2021; Ren et al. 2020).

The results showed noteworthy differences in the prediction performance of the obtained models. The higher performance quality of the ANN and BRT can be attributed to their capability to address the complexity of observed ecological mechanisms. This makes them well-suited for tackling these intricate problems (Hernandez‐Suarez and Nejadhashem 2018; Mata et al. 2021; Ren et al. 2020). The higher quality of both neural networks and boosted regression trees may be because they can deal with the complexity of the observed ecological mechanisms, making them suitable for these kinds of problems (Lemm et al. 2021; Park and Lek 2016). Both methods use iterative algorithms and obtain results by weighing the predictors in the learning or boosting procedures, making them suitable for extensive data analysis. The methods are relatively easy and fast to train and can produce highly accurate predictions of non-linear relationships and interactions between variables (El Bouchefry and de Souza 2020; Elith et al. 2008). Contrary to linear regression, they do not assume a linear relationship between the independent and dependent variables, the homogeneity of variances or the normality of data. For this reason, multilinear regression may not be appropriate for the considered problem because many studies indicate that the relationships observed in aquatic ecosystems are often highly complex and non-linear (Boldina and Meninger 2016), and new analytical method provides better results (e.g., Satish et al. 2022; Schreiber et al. 2022).

Early work (e.g. Silvert and Babtist 2000) pointed to the relatively small amount of ecological data available, which limited the application of ANNs. Similar inferences can be drawn for BRTs, taking into account their nature (Elith et al. 2008). However, the notable increase in monitoring efforts has guaranteed data availability for implementing new analysis approaches, making the adaptability of machine-learning techniques a reassuring aspect of the modelling process. Data obtained within water monitoring programs cope with different sources of bias (Schreiber et al. 2022), which predisposes using an ANN and BRT over MLR in modelling these relationships (Elith et al. 2008; Park and Lek 2016). Artificial neural networks have been considered a suitable alternative for modelling biological indices in rivers (e.g. Krtolica et al. 2021) and lakes (e.g. Luo et al. 2019). The BRT also indicated satisfactory quality in similar aquatic ecosystem modelling issues (Elias et al. 2016; Lemm et al. 2021).

One of the key aspects analysed in the study was the comparison of the modelling capabilities of two ecological assessment indices: IBMR and RVI. The results unequivocally indicated the higher modelling quality of the IBMR than that of the RVI, regardless of the applied model (ANN, BRT or MLR). This finding is crucial as it underscores the more consistent relation between the aquatic vegetation and the environmental variables (input data), particularly those with the most significant influence on the modelled values (Gebler et al. 2018). Additionally, linkages of macrophyte indices of river assessment, including the IBMR, with these factors were presented in other works (Aguiar et al. 2014; Krtolica et al. 2021), which confirms our findings. In fact, IBMR bioindicator species are truly aquatic species or hydrophytes and emergent species (or helophytes). These species rely heavily on water (hydrophytes) or are adapted to both wet and waterlogged substrates (helophytes). In addition, these aquatic communities are far more homogeneous than the riparian communities, which have complex vertical and spatial zonation within the riparian zone and a diverse linkage to the environment. The RVI incorporates aquatic and riparian vegetation, which can express the overall condition of rivers and streams and likely expresses the influences of multiscale environmental processes. However, the connection to environmental variables can be obscured by the diverse vegetation units (individual plants, vegetation patches, plant communities and riparian corridors) associated with specific spatial and temporal scales (González del Tánago et al. 2021). Some efforts have been made by Aguiar et al. (2011) to test the suitability of predictive modelling approaches for water quality assessment in Mediterranean rivers. They concluded that the performance of the diverse methods was difficult to compare as they express different types of disturbance acting at diverse spatial scales. Another limitation is the interannual variability affecting plant composition, which should also be incorporated.

Undoubtedly, there is a strong relationship between various environmental factors influencing aquatic ecosystems and riparian vegetation (Aguiar et al. 2011; González del Tánago et al. 2021; Rodrigues et al. 2019). However, the quality of our models revealed that this dependency cannot be utilised in the models. Based on this, we can also assume that our input variables did not include factors strongly associated with the RVI. Despite the evident connection between riparian vegetation and the river environment itself, the influence of other factors and stressors typical for terrestrial environments (e.g. soil characteristics), along with climatic factors, can significantly affect the riparian zones (Naiman and Décamps 1997). RVI is closely linked to the channel morphological changes and flow alterations, but with a span of multiple years, different from other variables. Stella and Bendix (2019) highlighted the numerous pressures exerted on riparian vegetation. These authors mentioned natural and anthropogenic disturbances more typical for terrestrial ecosystems (land use, storms and temperature), including factors relevant in the Mediterranean or similar climate ecosystems (fires and droughts). The higher quality of the modelling is likely attributed to the better relationship of the IBMR index to natural and anthropogenic factors determining the health of the aquatic environment. This index was designed explicitly for this purpose and has undergone extensive testing and calibration across various river types, including southern Europe (Haury et al. 2006; Aguiar et al. 2014; Stefanidis et al. 2022).

The variable importance measures (Wei et al. 2015) indicated that despite modelling two different indices using three different methods, comparable results were obtained regarding the most significant environmental factors influencing these indices. Important influences were attributed to longitude and geology, which can be associated with the specific characteristics of the studied rivers. In Portugal, the coast is primarily sedimentary (calcareous), highly affected by urban settlements and organic pollution and can affect aquatic vegetation (Aguiar et al. 2011). This factor was grouped with conductivity and alkalinity, factors often affecting river macrophyte development (Feio et al. 2012; Szoszkiewicz et al. 2020). Our research also suggested that hydrological conditions significantly influence macrophyte indices. The impact of water flow conditions in rivers (e.g. droughts and flash floods), especially in southern European rivers (Bonada and Resh 2013), was often highlighted as crucial for developing macrophytes and other aquatic organisms. This effect could be even more pronounced if temporary rivers were included in this study. These rivers have a large variability in hydrological conditions that greatly shape fluvial flora and fauna (Cid et al. 2017; Feio et al. 2012; Stefanidis et al. 2021, 2022). According to our results, other significant factors were water body size and land use, which can be linked to conditions within the catchment area, including pressures and their accumulation with the catchment area’s size and their direct impact on river ecosystems (Aguiar et al. 2011). These factors also appeared to be important factors affecting inland waters in the multistressor study (Lemm et al. 2021).

Among all the factors considered, the relevance of individual factors is quite similar for both indices, except for Factors 4 and 5, which show differences. Factor 4 is crucial for the IBMR (and not so important for the RVI), highlighting the significant impact of hydroclimatic conditions on macrophytes and the IBMR (Aguiar et al. 2014) compared to RVI. However, some works indicate the importance of this factor for riparian vegetation (Butterfield et al. 2023). In the second case, on the contrary, hydromorphological quality, also related to the quality of the river bed, but also the quality of banks and the riparian zone (Raven et al. 1998), had a greater influence on the riparian vegetation than the macrophyte in the river channel. This stands in contrast to other studies indicating a significant impact of hydromorphological quality on macrophyte development (e.g. Gebler et al. 2018) but could be due to a more significant influence of the hydrological regime on the populations and communities in the Mediterranean region (Cid et al. 2017). Some factors ranked lower in the importance analysis, such as total suspended solids and pH. Typically, these factors play a prominent role in plant distribution and growth, which has been demonstrated elsewhere (Demars et al. 2012). Comparable outcomes were achieved in neural network models for temperate rivers in Central and Northern Europe. In these models, factors other than pH, such as nutrients or habitat quality, were notably more significant (Bucior et al. 2021; Gebler et al. 2018; Krtolica et al. 2021; Szoszkiewicz et al. 2020). Interestingly, in the various models implemented for the IBMR, nutrients and habitat conditions were also not among the most significant factors, ranking fifth and sixth in importance, although they were often considered crucial for macrophyte development and differentiation (Haury et al. 2006; Szoszkiewicz et al. 2020), including macrophytes of the Iberian and Mediterranean rivers (Aguiar et al. 2014; Papastergiadou et al. 2016; Stefanidis et al. 2022).

The undervaluation of water physicochemical characteristics may be attributed to the extensive dataset employed in model development, which encompasses significant geographical variations across the country and shared ecological typologies that can bias the relevance of water quality variables. Notably, the dataset includes the longitudinal gradient (West‒East), mirroring geological distinctions, ranging from sedimentary coastal rivers in a high-populated area to continental rivers characterised by geological formations such as granites, schists and quartzites, each exhibiting diverse degrees of metamorphic alteration, modulated by varying altitude and climatic variables and less human pressure (Aguiar et al. 2009). Therefore, broad regional variables were more valued in the models and may obscure the local effects of nutrient loads from point sources (e.g. sewage) or nonpoint pollution sources (e.g. agriculture). From the management point of view, these findings indicate a need to refine the river typology and the pressure data inputs to overcome biogeographical differences.

## Conclusions

The presented study explored the potential of macrophyte-based indices to assess the water’s biological quality and the modelling methods for decision-making on river environmental management. Our findings prove that artificial neural networks and boosted regression trees are well-suited for modelling the intricate relationships between environmental variables and the plant biota of aquatic ecosystems. Almost all the developed models emphasised the crucial role of longitude and geology, indicating the importance of geographic factors (both anthropogenic and natural). Furthermore, the study revealed that in permanent rivers and streams of Southern Europe, hydrological conditions significantly impact macrophytes, surpassing the effect of nutrient levels or habitat quality. While the latter factors hold importance, they should be regarded as complementary factors in more comprehensive models.

## Data Availability

Data will be made available on request.

## Appendix

**Table 3:** Species of vascular hydrophytes and helophytes

Species	Family
*Alisma plantago-aquatica* L.	Alismataceae
*Antinoria agrostidea* (DC.) Parl.	Poaceae
*Apium nodiflorum* (L.) Lag.	Apiaceae
*Azolla filiculoides﻿﻿ *Lam.	Azollaceae
*Baldellia ranunculoides* (L.) Parl.	Alismataceae
*Bolboschoenus maritimus* (L.) Palla	Cyperaceae
*Callitriche brutia* Petagna	Callitrichaceae
*Callitriche hamulata* Koch	Callitrichaceae
*Callitriche stagnalis* Scop.	Callitrichaceae
*Carex elata* All*.* subsp*. reuteriana* (Boiss.) Luceño & Aedo	Cyperaceae
*Carex pendula* Hudson	Cyperaceae
*Ceratophyllum demersum* L.	Ceratophyllaceae
*Cirsium palustre* (L.) Scop.	Asteraceae
*Cyperus eragrostis* Lam.	Cyperaceae
*Cyperus esculentus* L.	Cyperaceae
*Cyperus longus* L.	Cyperaceae
*Eleocharis palustris* (L.) Roemer and Schultes	Cyperaceae
*Eleogiton fluitans* (L.) Link	Cyperaceae
*Eupatorium cannabinum* L.	Asteraceae
*Hydrocotyle vulgaris* L.	Apiaceae
*Hypericum elodes* L.	Clusiaceae
*Iris pseudacorus* L.	Iridaceae
*Isolepis setacea* (L.) Br.	Cyperaceae
*Juncus acutiflorus* Hoffm. subsp*. rugosus* (Steudel) Cout.	Juncaceae
*Juncus acutiflorus* Hoffm. subsp*. acutiflorus*	Juncaceae
*Juncus articulatus* L.	Juncaceae
*Juncus bulbosus* L.	Juncaceae
*Juncus heterophyllus* Dufour	Juncaceae
*Juncus subnodulosus* Schrank	Juncaceae
*Leersia oryzoides* (L.) Swartz	Poaceae
*Lemanea* sp.	Lemaneaceae
*Lemna gibba* L.	Lemnaceae
*Lemna minor* L.	Lemnaceae
*Litorella uniflora* (L.) Aschers	Plantaginaceae
*Ludwigia palustris* (L.) Elliot	Onagraceae
*Lycopus europaeus* L.	Lamiaceae
*Lysimachia vulgaris* L.	Primulaceae
*Lythrum salicaria* L.	Lythraceae
*Mentha aquatica* L.	Lamiaceae
*Myosotis secunda* A. Murray	Boraginaceae
*Myosotis stolonifera* (DC) Leresche & Levier	Boraginaceae
*Myriophyllum alterniflorum* DC.	Haloragaceaae
*Myriophyllum aquaticum* (Vell.) Verdc.	Haloragaceaae
*Myriophyllum spicatum* L.	Haloragaceaae
*Myriophyllum verticillatum* L.	Haloragaceaae
*Oenanthe crocata* L.	Apiaceae
*Phalaris arundinacea* L.	Poaceae
*Phragmites australis* (Cav.) Trin. ex Steud.	Poaceae
*Polygonum amphibium* L.	Polygonaceae
*Polygonum salicifolium* Brouss. ex Willd	Polygonaceae
*Pontederia crassipes Mart.*	Pontederiaceae
*Potamogeton crispus* L.	Potamogetonaceae
*Potamogeton natans* L.	Potamogetonaceae
*Potamogeton nodosus* Poiret	Potamogetonaceae
*Potamogeton polygonifolius* Pourr.	Potamogetonaceae
*Ranunculus flammula* L.	Ranunculaceae
*Ranunculus longipes* Lange ex Cutanda	Ranunculaceae
*Ranunculus ophioglossifolius* Vill.	Ranunculaceae
*Ranunculus peltatus* Schrank subsp.* peltatus*	Ranunculaceae
*Ranunculus trichophyllus* Chaix	Ranunculaceae
*Rorippa amphibia* (L.) Besser	Brassicaceae
*Rorippa nasturtium-aquaticum* (L.) Hayek	Brassicaceae
*Rorippa sylvestris* (L.) Besser subsp*. sylvestris*	Brassicaceae
*Schoenoplectus lacustris* (L.) Palla	Cyperaceae
*Scirpoides holoschoenus* (L.) Soják	Cyperaceae
*Scrophularia balbisii* Hornem. subsp. balbisii	Scrophulariaceae
*Sparganium erectum* L.	Sparganicaceae
*Stellaria alsine* Grimm	Caryophyllaceae
*Thalictrum flavum* L. subsp. *glaucum* (Desf.) Batt.	Ranunculaceae
*Typha dominguensis* (Pers) Steudel	Typhaceae
*Typha latifolia* L.	Typhaceae
*Veronica anagallis-aquatica* L.	Scrophulariaceae
*Veronica beccabunga* L.	Scrophulariaceae

**Table 4:** Species of bryophytes found in the active channel

Species	Family
*Amblystegium riparium* (Hedw.) Schimp.	Amblystegiaceae
*Atrichum undulatum* (Hedw.) P. Beauv.	Polytrichaceae
*Brachythecium plumosum* (Hedw.) Schimp.	Brachytheciaceae
*Brachythecium rivulare* Schimp.	Brachytheciaceae
*Brachythecium rutabulum* (Hedw.) Schimp.	Brachytheciaceae
*Bryum argenteum* Hedw.	Bryaceae
*Bryum capillare* Hedw.	Bryaceae
*Bryum pseudotriquetrum* (Hedw.) P. Gaertn., B. Mey. & Scherb	Bryaceae
*Calliergonella cuspidata* (Hedw.) Loeske	Hypnaceae
*Chiloscyphus polyanthos* (L.) Corda	Geocalycaceae
*Cinclidotus fontinaloides* (Hedw.) P. Beauv.	Cinclidotaceae
*Conocephalum conicum* (L.) Dumort.	Conocephalaceae
*Dialytrichia mucronata* (Brid.) Broth. var. mucronata	Pottiaceae
*Didymodon insulanus* (De Not.) M. O. Hill.	Pottiaceae
*Drepanocladus aduncus* (Hedw.) Warnst.	Campyliaceae
*Epipterygium tozeri (*Grev.) Lindb.	Bryaceae
*Eurhynchium hians* (Hedw.) Sande Lac.	Brachytheciaceae
*Eurhynchium praelongum* (Hedw.) Schimp.	Brachyteciaceae
*Eurhynchium praelongum* (Hedw.) Schimp. var*. stokesii* (Turner) Dixon	Brachyteciaceae
*Eurhynchium pulchellum* (Hedw.) Jenn.	Brachyteciaceae
*Fissidens bryoides* Hedw.	Fissidentaceae
*Fissidens bryoides* Hedw. var. caespitans Schimp.	Fissidentaceae
*Fissidens crassipes* subsp. warnstorffi (Fleisch.) Brugg.- Nann.	Fissidentaceae
*Fissidens polyphyllus* Wilson ex Bruch & Schimp.	Fissidentaceae
*Fissidens pusillus* (Wilson) Milde	Fissidentaceae
*Fissidens taxifolius* Hedw.	Fissidentaceae
*Fontinalis antipyretica* Hedw. var. *antipyretica*	Fontinalaceae
*Fontinalis hypnoides* Hartm. var. *duriaei* (Schimp.) Kindb.	Fontinalaceae
*Fontinalis squamosa* Hedw.	Fontinalaceae
*Fontinalis squamosa* Hedw. var. *dixonii (*Card.) A. J. E. Smith	Fontinalaceae
*Fossombronia angulosa* (Dicks.) Raddi	Fossombroniaceae
*Hygrohypnum ochraceum* (Wilson) Loeske	Campilaceae
*Hyocomium armoricum* (Brid.) Wijk & Marg.	Hypnaceae
*Isothecium holtii* Kindb.	Brachytheciaceae
*Leskea polycarpa* Hedw.	Leskeaceae
*Lunularia cruciata* (L.) Lindb.	Marchantiaceae
*Marchantia polymorpha* L.	Marchantiaceae
*Mnium hornum* Hedw.	Mniaceae
*Octodiceras fontanum* (Bach. Pyl.) Lindb.	Fissidentaceae
*Pellia endiviifolia* (Dicks.) Dumort.	Pelliaceae
*Pellia epiphylla* (L.) Corda	Pelliaceae
*Philonotis fontana* (Hedw.) Brid.	Bartramiaceae
*Plagiomnium undulatum* (Hedw.) T. J. Kop.	Mniaceae
*Plagiothecium nemorale* (Mitt.) A. Jaeger	Plagiotheciaceae
*Platyhypnidium lusitanicum* (Schimp.) Ochyra & Bednarek-Ochyra	Brachytheciaceae
*Pogonatum aloides* (Hedw.) P. Beauv.	Polytrichaceae
*Pohlia melanodon* (Brid.) Shaw	Mniaceae
*Polytrichum commune* Hedw.	Polytrichaceae
*Porella pinnata* L.	Porellaceae
*Racomitrium aciculare* (Hedw.) Brid.	Grimmiaceae
*Rhizomnium punctatum* (Hedw.) T. J. Kop.	Mniaceae
*Rhynchostegium riparioides* (Hedw.) Cardot	Brachytheciaceae
*Riccardia chamaedryfolia* (With.) Grolle	Aneuraceae
*Scapania undulata* (L.) Dumort.	Scapaniaceae
*Schistidium rivulare* (Brid.) Podp.	Grimmiaceae
*Scorpiurium deflexifolium* (Solms) M. Fleisch. & Loeske	Brachytheciaceae
*Sphagnum auriculatum* Schimp.	Sphagnaceae
*Thamnobryum alopecurum* (Hedw.) Gangulee	Neckeraceae

**Table 5:** The results of factor analysis

Variable	Factor 1	Factor 2	Factor 3	Factor 4	Factor 5	Factor 6	Factor 7	Factor 8
	Longitude and geology	Nutrients	Water body size	Hydrological/climatic conditions	Habitat quality	Land use	TSS and pH	Oxygen conditions
Latitude	− 0.404	0.102	0.129	0.809	− 0.100	0.134	0.110	0.013
Longitude	− 0.819	− 0.143	0.058	− 0.029	0.082	0.125	0.159	− 0.118
Altitude	− 0.400	0.123	0.481	0.350	0.052	0.443	− 0.059	0.032
Mean annual runoff	− 0.520	0.051	0.166	− 0.673	− 0.058	0.224	− 0.129	− 0.010
Catchment area	0.004	0.051	− 0.917	0.098	0.101	0.025	0.075	0.015
Distance to source	− 0.006	0.071	− 0.921	0.134	0.021	− 0.004	0.084	0.100
Thermal Range	0.089	− 0.088	− 0.263	0.487	− 0.122	0.007	0.251	− 0.199
Mean annual precipitation	− 0.546	− 0.012	0.178	− 0.702	0.033	0.177	− 0.087	− 0.040
Natural areas in the catchment	− 0.078	0.211	0.067	0.023	− 0.216	0.842	0.063	− 0.060
Artificial areas in the catchment	0.123	− 0.370	0.036	− 0.411	0.297	− 0.180	0.144	− 0.004
Extensive agriculture in the catchment	− 0.257	0.009	− 0.029	− 0.262	0.204	− 0.405	0.013	0.545
Intensive agriculture in the catchment	0.231	− 0.034	0.053	0.110	− 0.119	− 0.838	− 0.052	0.017
Riparian Forest Quality index	− 0.002	0.112	0.093	0.153	− 0.769	0.099	0.097	− 0.007
Habitat Quality Score	− 0.004	0.255	0.189	− 0.055	− 0.697	0.054	0.132	− 0.076
Habitat Modification Score	0.031	0.129	0.212	− 0.059	0.693	0.070	0.166	− 0.116
Water temperature	0.266	0.004	− 0.477	− 0.068	− 0.142	− 0.107	0.164	0.522
Dissolved oxygen concentration	− 0.332	0.203	0.049	− 0.079	0.076	− 0.082	− 0.030	− 0.779
pH	− 0.047	0.021	− 0.113	− 0.023	− 0.033	0.021	0.749	0.040
Conductivity	0.822	− 0.341	− 0.057	− 0.051	0.020	− 0.089	0.025	0.066
Alkalinity	0.763	− 0.146	0.056	0.039	0.119	− 0.061	0.129	0.080
Total suspended solids	0.084	− 0.001	− 0.057	0.276	− 0.002	0.062	0.784	0.041
Nitrate nitrogen	0.139	− 0.661	0.002	− 0.284	0.236	− 0.287	0.014	− 0.121
Ammonia nitrogen	0.071	− 0.833	0.046	0.033	− 0.006	0.019	− 0.062	0.130
Orto-phosphates	0.064	− 0.835	0.032	0.066	0.057	− 0.083	0.015	0.077
Cumulative variance (%)	19.23	33.35	42.46	50.10	56.35	62.19	66.98	71.39
